# Insights into the spatiotemporal dynamics of West Nile virus transmission in emerging scenarios

**DOI:** 10.1016/j.onehlt.2023.100557

**Published:** 2023-05-01

**Authors:** Laia Casades-Martí, Raúl Cuadrado-Matías, Alfonso Peralbo-Moreno, Sara Baz-Flores, Yolanda Fierro, Francisco Ruiz-Fons

**Affiliations:** a Instituto de Investigación en Recursos Cinegéticos (IREC), CSIC–UCLM–JCCM, Ciudad Real, Spain; b Yolfi Properties, Ciudad Real, Spain; c CIBERINFEC, ISCIII - CIBER de Enfermedades Infecciosas, Instituto de Salud Carlos III, Madrid, Spain

**Keywords:** Emerging diseases, Epidemiology, Flavivirus, Mosquito-borne diseases, Wildlife

## Abstract

The incidence of West Nile fever (WNF) is highly variable in emerging areas, making it difficult to identify risk periods. Using clinical case records has important biases in understanding the transmission dynamics of West Nile virus (WNV) because asymptomatic infections are frequent. However, estimating virus exposure in sentinel species could help achieve this goal at varying spatiotemporal scales. To identify the determinants of inter-annual variation in WNV transmission rates, we designed a 15-year longitudinal seroepidemiological study (2005–2020) in five environmentally diverse areas of southwestern Spain. We modeled individual annual area-dependent exposure risk based on potential environmental and host predictors using generalized linear mixed models. Further, we analyzed the weight of predictors on exposure probability by variance partitioning of the model components. The analysis of 2418 wild ungulate sera (1168 red deer - *Cervus elaphus* - and 1250 Eurasian wild boar - *Sus scrofa*) with a highly sensitive commercial blocking ELISA identified an average seroprevalence of 24.9% (95% confidence interval (CI): 23.2–26.7%). Antibody prevalence was slightly higher in wild boar (27.5%; CI: 25.1–30.1%) than in deer (22.2%; CI: 19.8–24.7%). We observed a spatial trend in exposure, with higher frequency in the southernmost areas and a slight, although area-dependent, increasing temporal trend. Host-related predictors were important drivers of exposure risk. The environmental predictor with the highest weight was annual cumulative precipitation, while temperature variations were also relevant but with less weight. We observed a coincidence of spatiotemporal changes in exposure with the notification of WNF outbreaks in horses and humans. That indicates the usefulness of wild ungulates as sentinels for WNV transmission and as models to understand its spatiotemporal dynamics. These results will allow the development of more accurate predictive models of spatiotemporal variations in transmission risk that can inform health authorities to take appropriate action.

## Highlights

• West Nile virus was circulating in Spain before West Nile fever emergence.
• Host- and weather-related predictors are drivers of WNV exposure dynamics.
• Antibody monitoring of wild ungulates is useful to study WNV transmission dynamics.
• Spatiotemporal dynamics of sentinel exposure to WNV shapes outbreak reporting.
• Predictive models based on sentinels may be useful to predict West Nile fever risks.

## Graphical abstract

**Figure:**
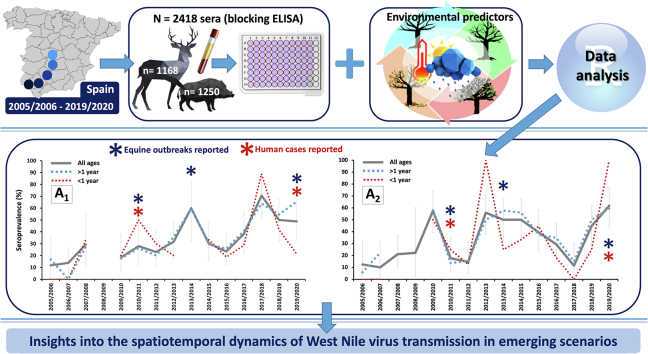

## 1 Introduction

The distribution of flaviviruses has increased considerably worldwide [1]. The vast majority of flaviviruses are vector-borne, and >50% of them are zoonotic and cause emerging diseases of variable animal and public health impact. Some flaviviruses of the Japanese encephalitis virus (JEV) serocomplex group, e.g., West Nile virus (WNV) or Usutu virus (USUV), have expanded their distribution range recently. This has favored the appearance of new West Nile fever (WNF) outbreaks and Usutu cases in livestock, humans, and wildlife [2]. *Flavivirus* infections often manifest clinically with signs of neuroinvasive disease that may progress to lethal disease [3]. In this regard, WNV and USUV are among the most impacting European flaviviruses [4]. However, JEV, Bagaza virus and other endogenous mosquito-borne flaviviruses have also been detected [[5], [6], [7]]. The flaviviruses of the JEV serocomplex are maintained by mosquitoes of the genus *Culex* and birds. However, their ecology is more complex and many other actors are involved in their local maintenance and transmission, e.g., abiotic environmental conditions and mammal presence/abundance [8,9].

In Spain, WNF emerged in horses, wild birds, and even humans at the beginning of the 21st century [2,10]. In 2007, WNV was confirmed as the cause of death of golden eagles (*Aquila chrysaetos*) in south-central Spain [11]. Neither human nor horse WNF case was reported until 2010 in the country [12]. Since then, the distribution area of WNV has expanded northwards. At present, lineages 1 and 2 are present in the southwest and northeast of Spain, respectively, with current incursions of both lineages in areas of the country distant from those in which they were initially detected. Two thousand and twenty was a remarkable epidemic year with 77 human WNF cases and eight casualties [2]. The short viremia and the high frequency of asymptomatic cases hinder early detection and favor the spread of WNV towards inland Iberia.

*Culex* spp. mosquitoes are widely distributed in Spain [13], and their remarkable adaptability to different environments, ranging from pond or reservoir waters to abandoned tires [14], makes them almost ubiquitous. In southern continental Spain, the most abundant *Culex* spp. are *C. pipiens* and *C. theileri* [15]. A female mosquito needs to feed on a viremic highly competent bird [4] or co-feed with an infected competent mosquito on a host [16] to become infected with WNV and transmit it while feeding again on a host. *Culex pipiens* feeds preferentially on birds but it also feeds on mammals, while *C. theileri* prefers mammals and can, eventually, feed on birds [14]. Likewise, the local abundance of *Culex* spp. and the transmission probability of WNV are shaped not only by weather conditions but also by host availability [17]. Wild ungulates experienced a demographic explosion in the Northern Hemisphere from mid 20th century [18] whose impact on mosquito dynamics is poorly understood. Wild ungulates are good WNV sentinels [8,9]. They would indeed be a better WNV monitoring group in Iberia when compared to birds because (1) they are widely distributed and abundant, (2) they are hunted in large numbers annually, and (3) their higher body size would potentially attract a higher number of mosquitoes as it occurs to ticks [19]. West Nile virus is the most detected *Flavivirus* in white-tailed deer [20] and feral pigs [21] in the USA, and also in Spanish wild ungulates [8,9]. Although the viremia they develop is insufficient for them to act as virus amplifiers, the serological reaction may be helpful to anticipate the occurrence of WNV outbreaks in areas of high risk of infection for susceptible hosts. No study has focused on unraveling WNV exposure dynamics determinants at small spatial scales. Thus, our goal was to use wild ungulates as WNV sentinels [8] and longitudinally study WNV exposure rate in the southwestern quarter of Spain to unravel virus dynamics in emerging areas of contrasting environmental favorability for its vectors. Wild ungulates could provide insights into the transmission ecology of WNV that would be helpful to progress in case prevention.

## 2 Materials and methods

### 2.1 Study design

**Fig. 1:**
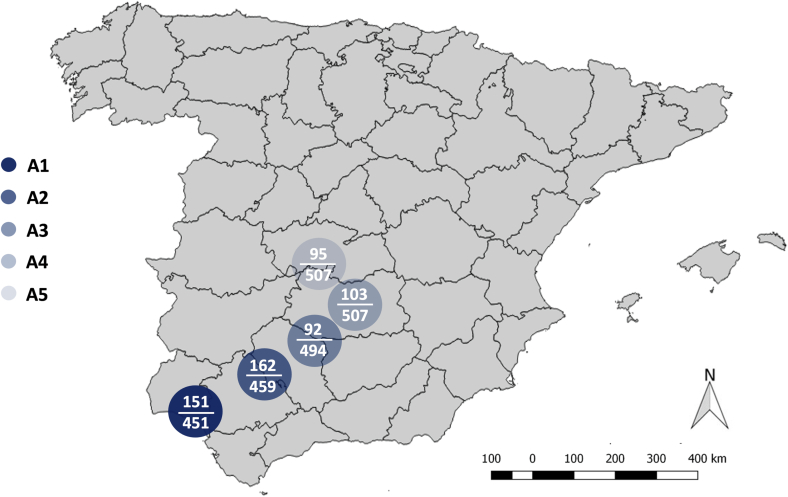
Allocation of the study areas (A_1_ to A_5_) to peninsular Spain. The number of bELISA positive samples (upper value) with respect sampling size (lower value) are displayed per study area.

Five areas in southwestern Spain were selected as representative of the wild ungulate populations at risk of exposure to WNV in the country (Fig. 1). The selected areas were (1) “Doñana” National Park (A_1_), (2) western “Sierra Morena” mountain chain (A_2_), (3) central “Sierra Morena” mountain chain (A_3_), (4) the “Guadiana River” valley (A_4_), and (5) the “Montes de Toledo” mountain chain. Climatic and environmental conditions are remarkably diverse in the study areas, especially in A_1_ if compared to the rest. In A_1_, a sub-humid thermo-Mediterranean climate with Atlantic influence predominates, where the average annual temperature is 17.6 °C and the average annual rainfall is 506.2 mm [22]. A_1_ habitat includes large areas of marshland, sand dunes, and maritime pine (*Pinus pinaster*) woodland patches interspersed within Mediterranean scrubland. In A_2_, A_3_, A_4_, and A_5_, the predominant habitat consists of a combination of woodland areas of holm oak (*Quercus ilex*), cork oak (*Q. suber*), and gall oak (*Q. faginea*) combined with pine (*Pinus* spp.) forests and abundant Mediterranean scrub (*Cystus* spp., *Rosmarinus* spp., *Erica* spp. and *Phillyrea* spp.) interspersed with patches of grassland. Average annual temperatures are 17.4 °C, 15.3 °C, 15.0 °C and 14.5 °C in A_2_, A_3_, A_4_, and A_5_, respectively; the average annual rainfall for the 2010–2019 period [22] in these areas was 670.6 mm, 585.5 mm, 570.1 mm, and 520.9 mm, respectively. In A_4_ and A_5_ the predominant climate is continental Mediterranean.

Several studies have shown exposure of wild birds to flaviviruses in A_1_ [23]. In both A_1_ and A_2_, WNF cases have been consistently reported in horses and humans since 2010 [12], and *Culex* spp. vectors are abundant [24]. WNF was confirmed in 2014 in a horse farm in A_4_ [25] and three positive birds were detected in 2007 in A_5_ [11]. Further, WNF has been reported in horses and birds to the north and west of A_5_ since 2014 [25]. *Culex pipiens* was recorded as the most abundant WNV vector by Durán-Martínez [15] in A_3_ to A_5_ followed in abundance by *C. theileri*. This evidence shows that the selected areas are appropriate for studying WNV emergence determinants in Spain.

We designed a longitudinal retrospective survey of WNV exposure in wild ungulates. We first calculated the sample size required to estimate annual seroprevalence per area. The estimation was based on the average antibody prevalence reported in wild ungulates in the region (4%) [8]. We adjusted the estimate for a large wild ungulate population (>2000 animals), with a precision of 7% and a confidence level of 95% [26]. Wild ungulate serum samples were collected in commercial hunting events annually in the study areas for 15 years. For ecological modeling purposes, the collection date was used to split samples into annual seasons (2005/2006 to 2019/2020). A season was defined as the period between April 1 in the year “*t*” and March 31 in the year “*t* *+* *1*”, so each season comprised the period of vector activity during which WNV can be transmitted [27]. In this way, we can ensure that a seropositive animal that was born in the spring of year “*t*” and sampled between April of that year and March of year “*t* *+* *1*” was exposed to WNV during the mosquito activity season of its birth year.

Wild ungulates harvested by hunters, gamekeepers, or environment agents in commercial hunting events or population control trials were surveyed from 2005 to2020 (Table 1). Sampling was conducted in accordance with Spanish and EU regulations. Sera collected from 2418 animals (1168 red deer – *Cervus elaphus* – and 1250 Eurasian wild boar – *Sus scrofa*) were selected. Data on spatial location, municipality, and survey date were recorded. Animals were sexed and their age was estimated through tooth eruption patterns [28] and split into (1) yearling (<1 year old), (2) juvenile (1–2 years old), and (3) adult (>2 years old) age classes.

**Table 1:** Number of collected and analyzed wild ungulate serum samples throughout the 15 survey seasons and the five study areas. *NA*: Samples were not available.

Survey season	A_1_	A_2_	A_3_	A_4_	A_5_	*Total*
2005/2006	17	24	34	19	28	*122*
2006/2007	51	20	34	48	35	*188*
2007/2008	17	38	30	33	34	*152*
2008/2009	*NA*	9	34	34	32	*109*
2009/2010	27	33	32	32	38	*162*
2010/2011	25	34	32	35	27	*153*
2011/2012	35	34	35	33	35	*172*
2012/2013	41	34	34	34	40	*183*
2013/2014	15	34	28	34	28	*139*
2014/2015	33	34	34	33	40	*174*
2015/2016	47	33	34	35	34	*183*
2016/2017	34	34	34	34	34	*170*
2017/2018	34	26	34	34	28	*156*
2018/2019	34	38	32	44	40	*188*
2019/2020	41	34	33	25	34	*167*
***Total***	***451***	***459***	***494***	***507***	***507***	***2418***

### 2.2 Laboratory analyses

Multi-species blocking enzyme-linked immunosorbent assays (bELISA; INGEZIM West Nile COMPAC®, Ingenasa, Madrid, Spain) were performed to the selected sera for the detection of WNV antibodies following the manufacturer's recommendations. This commercial test is the most specific for WNV of all commercial WNV serological assays [29]. The bELISA displays high sensitivity (100%) and good specificity (79.5–96.5%) for anti-WNV anti-NPV antibodies [30].

### 2.3 Weather determinants

The population dynamics of WNV vectors, and thus of WNV transmission dynamics, is modulated by abiotic environmental conditions [31]. Unusual warm winters or rainy years could favor vector populations locally and maximize virus transmissibility. Therefore, stochastic variations in local weather conditions, rather than prevailing climatic conditions, were better suited to the objectives of our study. Meteorological parameters were collected from 2004 to 2020 from weather stations managed by the Spanish Meteorological Agency (AEMET). Different variables related to temperature and rainfall were estimated (Table 2). Each individual animal was related to the estimated data from the spatially closest meteorological station. Annual mean values of the meteorological magnitudes were calculated from monthly records and taking into account the sampling season described above (April 1 to March 31).

**Table 2:** Set of predictors (within four factor types) estimated for statistical analyses and their range values. The predictors selected for inclusion in multiple risk factor modeling are shown in bold.

Factor	Predictor	Description	Type & values
Spatial	**sa**	Survey area	Categorical (1–5)
Temporal	**s**	Season	Categorical (1–15)
Host	**sp**	Wild ungulate species	Categorical (1–2)
	**ag**	Age class	Categorical (1–3)
Abiotic	**ar**	Annual accumulated rainfall	Continuous (144.3–1581.9 mm)
	swr	Rainfall accumulated over Dec-May	Continuous (49.8–1363.6 mm)
	**sr**	Spring (March–May) accumulated rainfall	Continuous (6.3–608.9 mm)
	**smr**	Summer (July-Sept) accumulated rainfall	Continuous (0.0–128.0 mm)
	**wt**	Average winter (Dec-Feb) temperature	Continuous (4.5–12.3 °C)
	**st**	Average spring (March–May) temperature	Continuous (10.4–18.0 °C)
	**smt**	Average summer (July-Sept) temperature	Continuous (17.0–28.0 °C)

### 2.4 Statistical analyses

To avoid common problems in data modeling, a comprehensive descriptive analysis was performed as recommended by Zuur et al. [32] and some highly correlated variables were excluded (Table 2). All the continuous variables were rescaled prior to modeling to homogenize the scales of measures by applying natural logarithmic transformations. Once the exploratory analysis of the data was performed, the selected variables were included in all possible combinations of linear generalized mixed models considering survey area and year (season) as random factors. Models were ranked according to the corrected Akaike information criterion (AICc), using the ‘dredge’ function of the MuMin R package [33], and models with an AICc difference (ΔAICc) of less than two were included in the model averaging process to obtain the final best-fit model [see [34]]. We analyzed multicollinearity effects by estimating the variance inflation factor for model parameters [35]. A final step was to estimate the relative contribution of each model parameter to the variation of the probability of exposure. For this, we partitioned the coefficient of determination *R*^2^ of the predictors included in the best model by estimating the semi-partial (part) *R*^2^ and the structural coefficients with the R package ‘partR2’ [36].

## 3 Results

### 3.1 Descriptive findings

The sample size required per season and study area was 31, so we adjusted available samples in our sera bank to this number whenever possible. Sample selection was performed to balance sex and age classes in the study spatiotemporal scale unit (see Table 3). Six hundred and twenty-six of the 2418 animals (266 deer and 360 wild boar) were less than one year old, 735 (329 deer and 406 wild boar) were between one and two years old, and 957 (551 deer and 406 wild boar) were more than two years old. Sex was recorded in 2312 animals (1129 males and 1183 females), of which 1140 were red deer (574 males and 566 females) and 1172 were wild boar (555 males and 617 females).

**Table 3:** Summary of population (p) and yearling (y) specific bELISA results (No. of bELISA positive vs. the no. of sera analyzed, and the proportion of seropositive animals - in % - within brackets) throughout study area along the 15-year study period. Data are split for wild ungulates (WU: red deer + Eurasian wild boar), red deer (RD) and Eurasian wild boar (WB).

Area	WUp NP/NT (P)⁎	WUy NP/NT (P)	RDp NP/NT (P)	RDy NP/NT (P)	WBp NP/NT (P)	WBy NP/NT (P)
A_1_	151/451 (33.3%)	33/112 (29.5%)	61/196 (31.3%)	7/32 (31.9%)	90/255 (35.3%)	26/80 (32.5%)
A_2_	162/459 (35.3%)	24/82 (29.3%)	70/207 (33.8%)	9/26 (34.6%)	92/252 (36.5%)	15/56 (26.8%)
A_3_	92/494 (18.6%)	17/130 (13.1%)	43/259 (16.6%)	11/63 (17.5%)	49/235 (20.9%)	6/67 (9.0%)
A_4_	103/507 (20.3%)	23/138 (16.7%)	38/253 (15.0%)	11/66 (16.7%)	65/254 (25.6%)	12/72 (16.7%)
A_5_	95/507 (18.7%)	23/164 (14.0%)	47/253 (18.6%)	10/79 (12.7%)	48/254 (18.9%)	13/85 (15.3%)
*Total*	*603/2418* *(24.9%)*	*120/626* *(19.2%)*	*259/1168* *(22.2%)*	*48/266* *(18.0%)*	*344/1250* *(27.5%)*	*72/360* *(20.0%)*

⁎ Number of positive (NP) and tested (NT) samples, and estimated antibody prevalence (P) within brackets.

In total, 603 of the 2418 animals were antibody positive (24.9%; 95% confidence interval (CI): 23.2–26.7%). Considering the specificity of the test (79.5%–96.5%), seroprevalence values would be between 19.8% and 24.1%. Of these, 259 were deer (259/1168, 22.2%; CI: 19.8–24.7%), and 344 were wild boar (344/1250, 27.5%; CI: 25.1–30.1%). Based on the specificity of the test, this would result in an antibody prevalence ranging 17.6%–21.4% for red deer and 21.9%–26.6% for wild boar. The highest seroprevalence values were found in A_2_ (35.3%; CI: 30.9–39.9%), followed by A_1_ (33.5%: CI: 29.1–38.1%), A_4_ (20.3%: CI: 16.9–24.1%), A_5_ (18.7%: CI: 15.4–22.4%) and A_3_ (18.6%: CI: 15.3–22.3%), respectively (see Fig. 1). In all areas, seroprevalence values were higher in wild boar than in red deer (see Table 3).

### 3.2 Spatiotemporal trends in seroprevalence

**Fig. 2:**
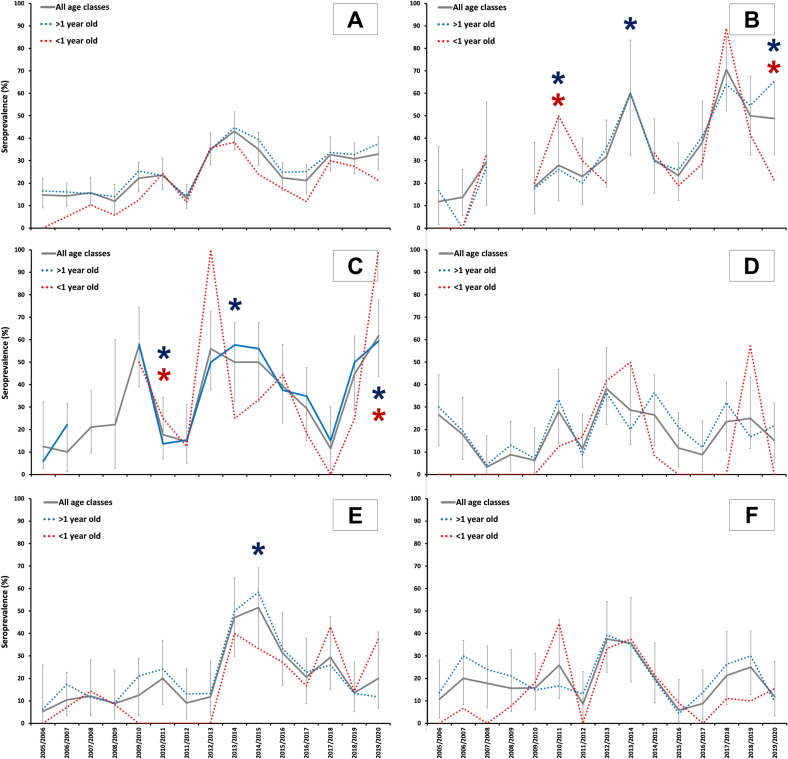
Evolution of WNV antibody prevalence in wild ungulates (red deer and wild boar) in all study areas (A) and in A_1_ (B), A_2_ (C), A_3_ (D), A_4_ (E) and A_5_ (F). The grey solid line shows population seroprevalence and associated 95% exact confidence interval of estimates). The blue dotted line shows seroprevalence in >1 year-old animals. Finally, the red dotted line shows seroprevalence values for yearlings (<1 year old). Blue asterisks represent years with remarkable WNF equine outbreaks and brown ones represent those years in which human cases were reported. (For interpretation of the references to colour in this figure legend, the reader is referred to the web version of this article.)

Exposure to WNV was observed during the study period in all study areas (Fig. 2). A WNV transmission peak was observed in 2013/2014 with antibody prevalence reaching 43.2%. However, the temporal exposure dynamics was locally variable. In A_1_, two prevalence peaks were observed in 2013/2014 and in 2017/2018, both with rates above 60%. The prevalence peaks in A_2_ were of similar levels to those in A_1_, with two peaks observed in 2009/2010 and 2019/2020 and a period of high prevalence between 2013/2014 and 2015/2016 with respect to the average values of the whole study period in this area. These peaks in A_1_ and A_2_ occurred mainly in years when numerous outbreaks in equids and human cases of WNF were declared in southwestern Spain [25] (Fig. 2). In several years we observed a temporal coincidence between incidence peaks in animals less than one year of age in A_1_ and A_2_ and the notification of WNF cases. In A_3_, several minor prevalence peaks (2010/2011, 2012/2013 and 2017/2018 to 2018/2019) were observed in the population with values around 30–40%. In A_4_, the temporal evolution of WNV exposure was more stable with a peak in prevalence and incidence between 2013/2014 and 2014/2015 that coincided with the notification of the only documented case of WNF in equids in this area. Finally, the exposure evolution scenario in A_5_ was stable, with a peak prevalence around 40% in 2012/2013 to 2013/2014 in which we also observed a notable increase in estimated incidence with respect to the previous season, and a peak of lower prevalence (around 20%) between 2017/2018 and 2018/2019.

Increasing seroprevalence patterns were observed in the core study region and in areas A_1_, A_2_, and A_4_ in contrast to the detrended exposure pattern observed in A_3_ and A_5_. Disregarded local variations in exposure trends, seroprevalence local patterns displayed high disparity between contiguous years. Population seroprevalence patterns evolved over time in a manner very similar to the variation in the estimated incidence of new cases in animals <1 year of age (Fig. 2). Seasonal prevalence and incidence peaks did indeed temporarily match in most of the seasons and areas.

### 3.3 Exposure risk determinants

**Fig. 3:**
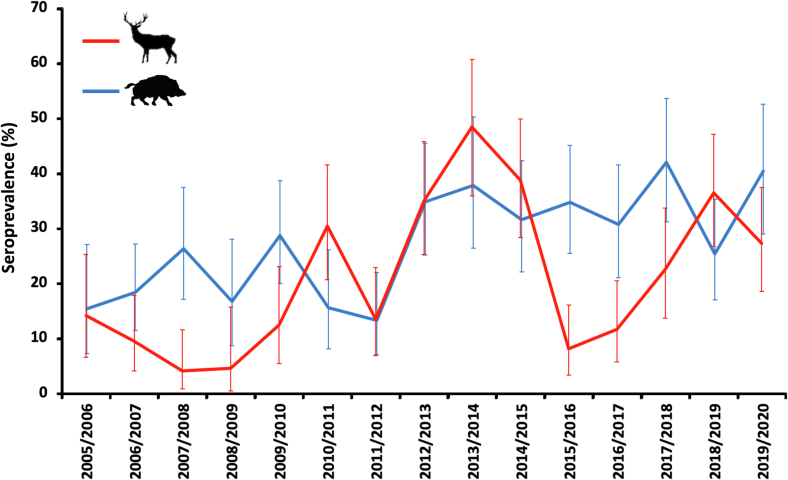
Evolution of WNV antibody prevalence (and associated 95% exact confidence intervals) per study season (2005/2006 to 2019/2020) throughout wild ungulate species. Red line is used for red deer and blue line for wild boar. (For interpretation of the references to colour in this figure legend, the reader is referred to the web version of this article.)

**Fig. 4:**
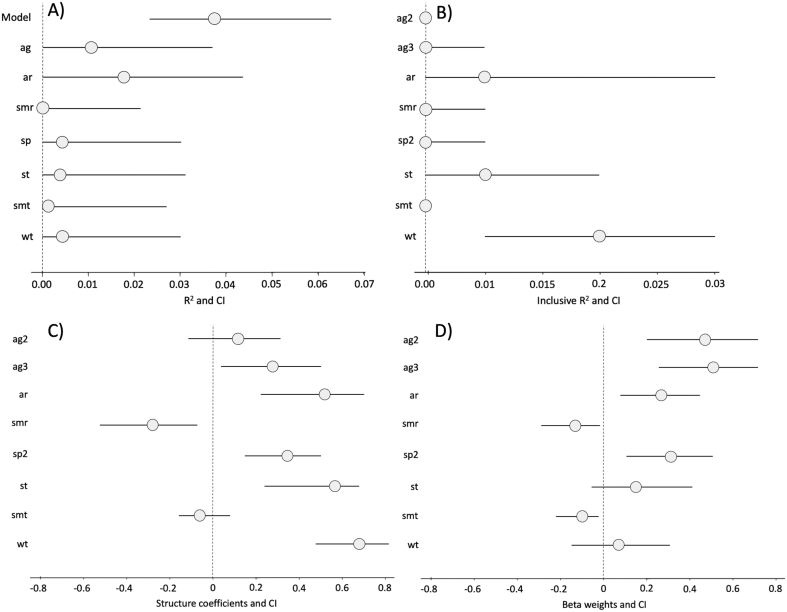
Forest plots for comparison of part R^2^ (coefficient of determination) for model predictors (A), inclusive R^2^ (B), structure coefficients (C) and beta weights (C) including confidence intervals (CI) for the general population risk model. The acronyms for each predictor are included as described in Table 2.

Ten models were best fitted to explain variation in WNV exposure probability (ΔAICc<2; Supplementary Table 1). The resulting average model included: 1) individual host predictors such as age and host species; and 2) weather predictors. Statistically significant host-realted predictors included age class and species. This revealed a higher probability of WNV exposure in >1 year-old animals relative to yearlings, and a higher risk in wild boar than in red deer (Fig. 3). Annual and summer accumulated rainfall were both also selected as relevant modulating predictors. An overall positive association was observed with increasing annual rainfall whereas exposure probability was higher in the driest summers (Table 4). The largest risk weights for individual predictors were age and annual rainfall (Fig. 4). Although less relevant, temperatures were also selected as risk-modulating predictors in the best-fitted model.

**Table 4:** Output of average selected model including the selected predictors (Acronyms are shown in Table 2), the estimate and its associated standard error (SE), the statistic (z) and the *p*-value.

Predictor	Estimate	SE	z	p
*Intercept*	*−3.82000*	*4.94098*	*0.773*	*0.439601*
ag				
yearling	Ref.	–	–	
juvenile	0.45208	0.1363	3.276	***
adult	0.50669	0.13105	3.866	***
ar	0.57407	0.23221	2.471	*
smr	−0.12395	0.05601	2.212	*
sp				
red deer	Ref.	–	–	
wild boar	0.33185	0.10178	3.259	**
smt	−0.75666	1.48061	0.511	0.609409
wt	0.12208	0.35278	0.346	0.729372
st	0.36469	0.80808	0.451	0.651859

*p < .05; **p < .01; ***p < .001.

## 4 Discussion

Our study reconfirms the presence of WNV antibodies among Iberian wild ungulate populations using a highly sensitive blocking ELISA test as reported by Boadella et al. [8], García Bocanegra et al. [9], and Caballero-Gómez et al. [37] in Spain. No study has previously dealt with unraveling the determinants of the small spatial scale transmission dynamics of WNV in a longitudinal approach, so our findings are novel and relevant to understanding and better predicting local transmission risks for improved prevention.

The observed seroprevalence is above the one previously reported in wild ungulates in Spain [8,9] and in other countries [20,38,39]. Considering that the first cases of WNF occurred in 2010, it is noteworthy that in all our 15 years of study, the seroprevalence was above 12%. The higher mean antibody prevalence observed in our study compared to those reported previously is probably related to the increasing environmental favorability for WNV vectors with time as the spread of WNV in the region indicates. In fact, Boadella et al. [8] observed a 10-fold increase in the seroprevalence in this region after the notification of the first WNF outbreaks in 2010, indicating the clear expansion of the virus that we confirm in this study and that the remarkable increase in WNF notifications throughout Spain corroborates [25]. The early detection in time of WNV antibodies in our study is consistent with the detection of antibodies after 2004 in wild birds [23]. It also agrees with reports from raptors in 2007 [11] and with WNV-positive mosquitoes in 2008 and 2009 [40]. Altogether, these findings confirm the active circulation of WNV in southern Spain well in advance of the first outbreaks in equine farms. Further, the seroprevalence in areas A_3_ to A_5_ from the 2005/2006 to 2009/2010 seasons ranged between 3 and 27% (see Fig. 2). This indicates that WNV may have been prevalent before outbreak reporting. Most probably, sporadic WNF cases went unnoticed by veterinary and medical practitioners as it occurred with Crimean-Congo hemorrhagic fever virus in the country [41]. Our results do not show a steep increasing trend in exposure as was observed with other emerging infections, e.g., bluetongue virus [42]. However, the time trend was positive in those areas where WNF outbreaks were reported (A_1_, A_2_, and A_4_), which may indicate that changes in environmental conditions have favored the emergence of WNF. In fact, winter temperatures show an increasing trend over the study period in these areas (see Supplementary Fig. 1), which may have favored vector survival rates in winter (perhaps also of WNV avian reservoirs) and resulted in an increase in transmission.

Boadella et al. [8] reported higher seroprevalence values in wild boar when compared to red deer. Several hypotheses could aid in explaining this finding that was also observed in our study. Wild boar prey on small animals and scavenge on the carcasses of various species [43], including birds. Several birds, e.g., waterfowl, corvids, or raptors, are highly sensitive to WNV infection [44] and their infected carcasses could be serving as wild boar food. Alternative hypotheses include potential differences in host selection preferences of WNV vectors, differences in space use between red deer and wild boar, or even differences in their daily activity patterns in relation to the host-seeking activity patterns of WNV vectors. The increasing pattern of exposure risk with age is in line with expectations because the risk of interacting with an infected mosquito increases with lifetime and WNV antibodies may persist for >5 years in mammals [45]. However, even though an expected long persistence of WNV antibodies, the evolution of seroprevalence in yearlings (indicators of exposure in the sampling year) and in the population (Fig. 2) overlapped, indicating that annual variations in exposure frequency occur in the overall population and that annual exposure rates can be considered as good indicators of infection incidence. Therefore, modeling the annual risk on the population in a particular year would be indicative of the real risk of exposure in this year. This could be, within an emerging scenario, the consequence of large inter-annual variations in environmental conditions that generate yearly variations in vector abundance together with the low average WNV antibody prevalence (when compared to other vector-pathogen models such as *Hyalomma* spp. ticks and Crimean-Congo hemorrhagic fever virus [46]).

The observed spatial seroprevalence pattern agrees with the highest number of outbreaks being reported in A_1_ and A_2_ [25]. This could be a consequence of differences in environmental conditions triggering variations to the burdens of mosquito vectors as well. In this regard, the diversity and abundance of *Culex* spp. are much lower in zones A_3_, A_4_ and A_5_ [15,47] where a dry continental climate predominates, compared to those reported in the two sub-humid thermo-Mediterranean climate zones with Atlantic influence, A_1_ and A_2_ [24,48]. This fact can be clearly linked to the positive influence of the accumulated rainfall on the risk of exposure to WNV, especially relevant in inland dry continental areas of Spain. The rainfall regime, unlike temperatures, did not show a clear trend during the study period in any of the areas (Supplementary Fig. 1), but a very changeable pattern between years as is usual in a Mediterranean climate. The rainfall filling semi-permanent or seasonal ponds with water is crucial for egg laying and development of mosquito larval stages, and thus a relevant parameter on their abundance [14], consequently resulting in a higher risk of WNV transmission. However, when water availability does not depend directly on the local rainfall regime but on that of its hydrographic basin, as is the case of A_1_, the dependence of these will be less in comparison with non-aquatic ecosystems such as those of the other four study areas. Although being a relevant modulating factor for vector populations, the model did not indicate a significant effect of temperature on risk even though different temperature-related parameters were selected within the best-fitted model. Differences between average winter and summer temperatures do not exceed 13 °C in A_1_ whereas those differences are over 18 °C in areas A_3_ to A_5_. The more frequent freezing winter conditions in continental Spain (see supplementary Fig. 1) could reduce overwintering survival rates of *Culex* spp. and be behind the differences in vector abundance. The increasing temperature pattern that we observed in continental Spain in the study period suggests that a positive change in the environmental favorability for WNV vectors is happening. However, the relevance of the local rainfall regime indicates that both parameters jointly modulate WNV vector population dynamics, what should be explored in more detail in the future. Further, spring temperatures are more appropriate for mosquito reproduction in A_1_ and A_2_ whereas summer temperatures are extremely high in all areas except in A_1_. The combination of high annual average rainfall (A_2_) or surface water accumulation (A_1_) and a warmer spring would render more favorable environmental conditions for mosquitoes to breed and transmit WNV in comparison to the rest of the study areas.

The temporal patterns of variation in WNV exposure in our study neatly aligned with the record of equine and human outbreaks since 2010 in or near these areas. In 2010, 36 outbreaks in horses and two human cases were recorded in southwestern Spain [2,25], coinciding with the seroprevalence peaks observed in 2009/2010 and 2010/2011 in areas A_1_ and A_2_. In 2013, 35 outbreaks in equines were recorded in the same area, and again we observed a marked increase in exposure in A_1_ and A_2_. In area A_4_, the only equine outbreak was recorded in 2014, when we observed the highest seroprevalence level in the area. Further, between 2018 and 2020, we observed high seroprevalence levels in A_1_ and A_2_. In 2020, 136 equine outbreaks and 77 human cases were reported in southern Spain [2,25]. This finding suggests that monitoring WNV exposure evolution in wild ungulates (commercially hunted and the subject of the Spanish national wildlife health surveillance program) could alert to the potential occurrence of WNF outbreaks. The combination of wild ungulate monitoring with wild bird active and passive surveillance, livestock monitoring, and mosquito surveillance would significantly improve our chances to predict the risk of WNF emergence in specific areas of Spain and thus reduce its impact. Our goal was not to predict spatiotemporal risks, but our findings highlight the usefulness of building predictive models based on sentinels to improving preventive capabilities against this disease.

## 5 Conclusions

West Nile virus was circulating in Spain long before the emergence of WNF. The main determinants of its transmission, excluding host-related predictors, are environmental and probably mostly related to vector abundance. We reconfirm the usefulness of wild ungulates as sentinels to monitor WNV transmission dynamics, but recommend that an integrated WNV surveillance program including other wildlife species, domestic animals and vectors that are relevant determinants of WNV ecology is designed and implemented in the framework of an expected increase in WNF incidence in Spain in the near future.

## Funding

This study was funded by the Spanish Ministry for the Science and Innovation (MCI) through the Spanish Research Agency (AEI) and EU FEDER (*E*-RTA2015-0002-C02 & CGL2017-89866-R grants), and by the Regional Government of Castilla-La Mancha (JCCM) and the 10.13039/501100004895European Social Fund (ESF) (SBPLY/19/180501/000321 grant). LC-M, RC-M, AP-M and SB-F acknowledge the support of MCI, 10.13039/501100011698JCCM, 10.13039/501100004895European Social Fund (ESF), and the 10.13039/501100007480University of Castilla-La Mancha (UCLM) through grants PEJ2018-003155-A, PRE2018-083801, 2019-PREDUCLM-10932 and PREJCCM2019/11.

## Ethics statement

The study was conducted on red deer and wild boar that were shot by hunters during commercial hunting events or by environment agents as part of population control measures. Therefore, they were not shot deliberately for this study and no ethical permission was required to gather samples.

## CRediT authorship contribution statement

**Laia Casades-Martí:** Conceptualization, Data curation, Formal analysis, Investigation, Writing – original draft, Writing – review & editing. **Raúl Cuadrado-Matías:** Data curation, Investigation, Writing – review & editing. **Alfonso Peralbo-Moreno:** Data curation, Investigation, Writing – review & editing. **Sara Baz-Flores:** Data curation, Investigation, Writing – review & editing. **Yolanda Fierro:** Data curation, Writing – review & editing. **Francisco Ruiz-Fons:** Funding acquisition, Conceptualization, Data curation, Formal analysis, Investigation, Writing – original draft, Writing – review & editing.

## Declaration of Competing Interest

The authors declare no conflict of interest.

## Data Availability

Data will be made available on request.

## Appendix A Supplementary data

Supplementary material Image 1
