# Supplementary material for: Insights into the spatiotemporal dynamics of West Nile virus transmission in emerging scenarios
Source: One Health. 2023 May 1;16:100557. doi: 10.1016/j.onehlt.2023.100557 (PMC10288089; doi:10.1016/j.onehlt.2023.100557)

**Supplementary Table 1.** Set of models with ΔAICc < 2 selected for model averaging. Predictor abbreviations are defined in Table 2 of the paper.

| **Model reference** | **ΔAICc/weight** | **Predictors** | | | | | |  |
| --- | --- | --- | --- | --- | --- | --- | --- | --- |
|  |  | **ar** | **smr** | **wt** | **st** | **smt** | **sp** | **ag** |
| model 1 | 0.00/0.32 | 0.5583 | -0.1287 | ns | ns | ns | + | + |
| model 2 | 1.02/0.19 | 0.5333 | -0.1236 | ns | ns | -1.717 | + | + |
| model 3 | 1.18/0.18 | 0.5499 | -0.1230 | 0.6951 | ns | ns | + | + |
| model 4 | 1.31/0.16 | 0.6437 | -0.1133 | ns | 1.4460 | -2.617 | + | + |
| model 5 | 1.45/0.15 | 0.6103 | -0.1272 | ns | 0.8288 | ns | + | + |

ns: predictors not included in models with ΔAICc < 2.

**Supplementary Figure 1.** Yearly variation in accumulated annual rainfall (left charts) and average winter temperature (Dec-Feb; right charts) per study area (A_1_: “Doñana” National Park; A_2_: western “Sierra Morena” mountain chain; A_3_: central “Sierra Morena” mountain chain; A_4_: the “Guadiana River” valley; and A_5_: the “Montes de Toledo” mountain chain) in relation to the evolution of WNV antibody prevalence in the population of wild ungulates (grey line) along fifteen sampling seasons (2005/2006 to 2019/2020).


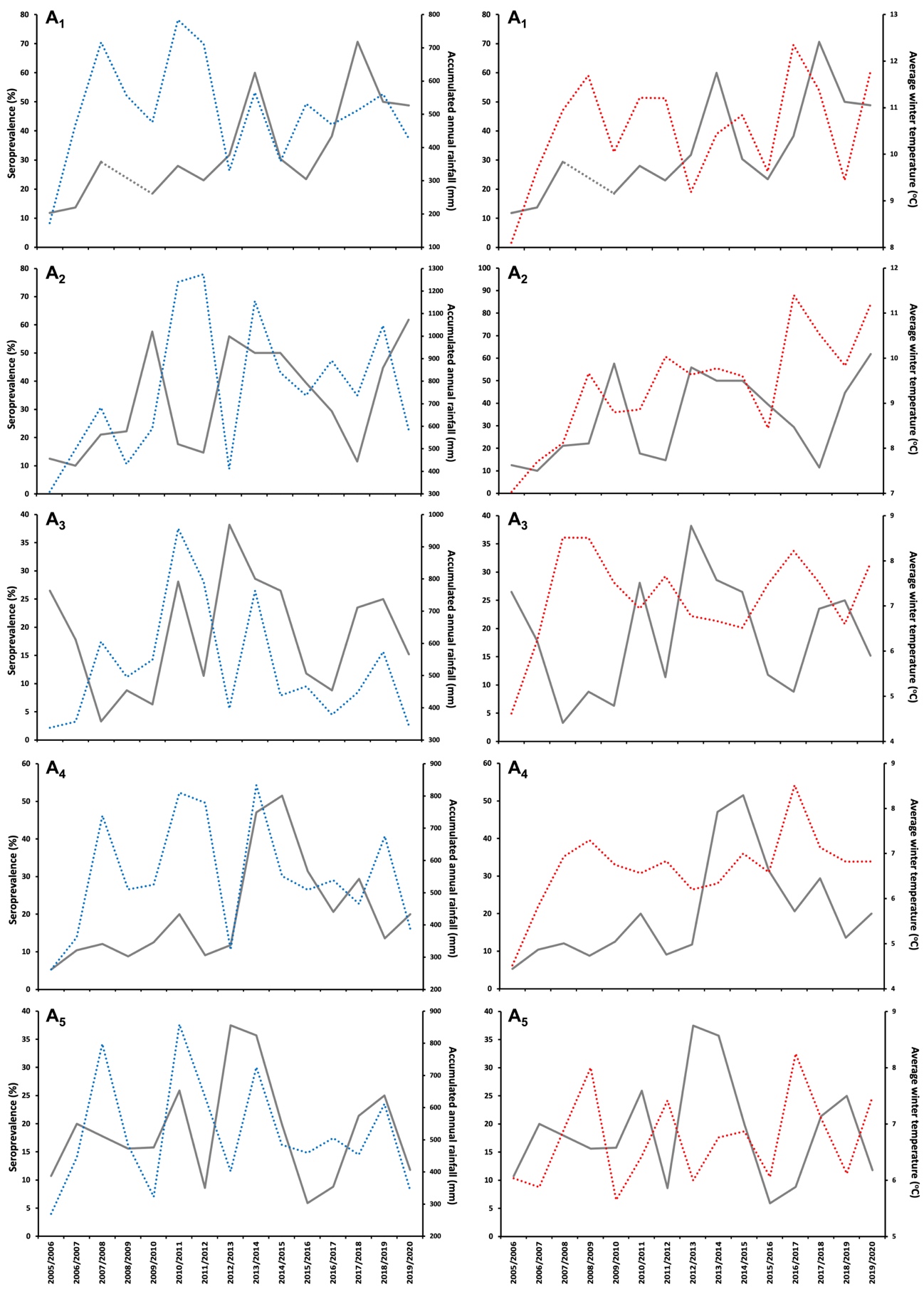

Supplement: Supplementary file 1 — Supplementary material [file mmc1.docx]
